# Supplementary material for: Integrative Analysis of Single-Cell and Bulk Sequencing Data Depicting the Expression and Function of P2ry12 in Microglia Post Ischemia–Reperfusion Injury
Source: Int J Mol Sci. 2023 Apr 5;24(7):6772. doi: 10.3390/ijms24076772 (PMC10095011; doi:10.3390/ijms24076772)
Supplement: Supplementary file 1 [file ijms-24-06772-s001.zip › ijms-2265212-supplementary.pdf]

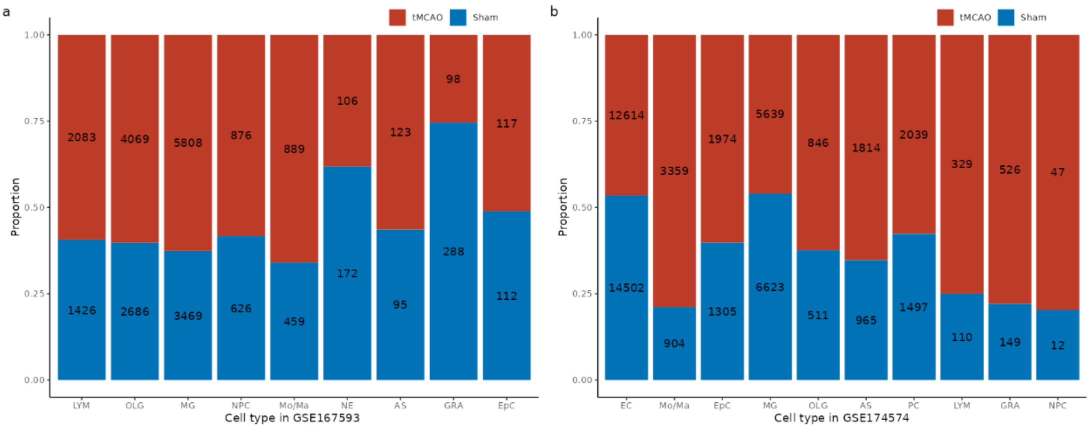

Figure S1. Details of the demographic characteristics in scRNA-seq data. (**a**) and (**b**) Column plots show cell number and proportion of each cell type from different groups in GSE167593 and GSE174574, respectively.

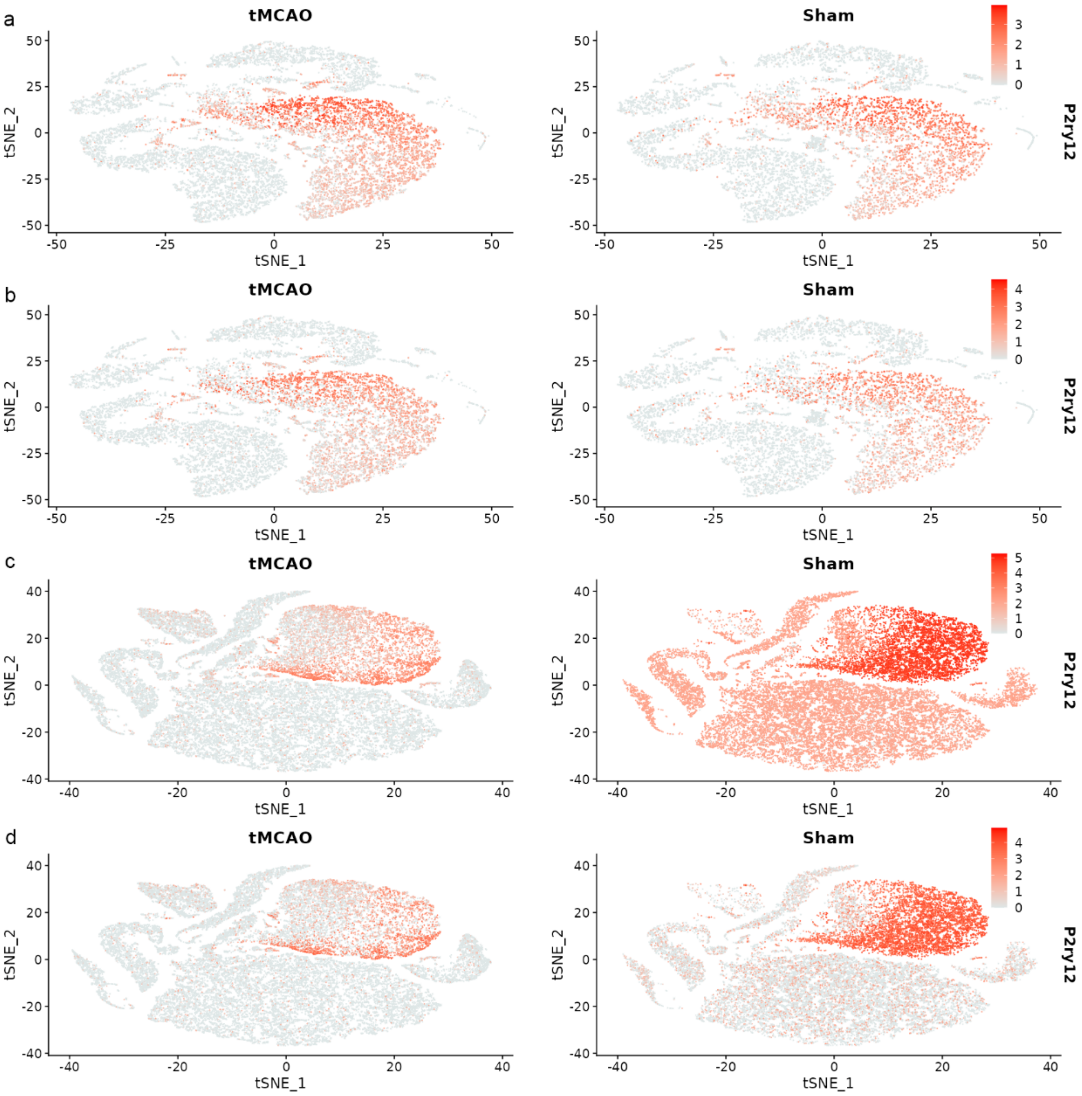

Figure S2. Distribution of P2ry12 Gene Expression in Single-Cell RNA-Seq Data. (**a**) t-SNE visualization of P2ry12 expression in imputed GSE167593 dataset. (**b**) t-SNE representation of P2ry12 expression in pre-imputed GSE167593 dataset. (**c**) t-SNE representation of P2ry12 expression in imputed GSE174574 dataset.

(**d**) t-SNE representation of P2ry12 expression in pre-imputed GSE174574 dataset.

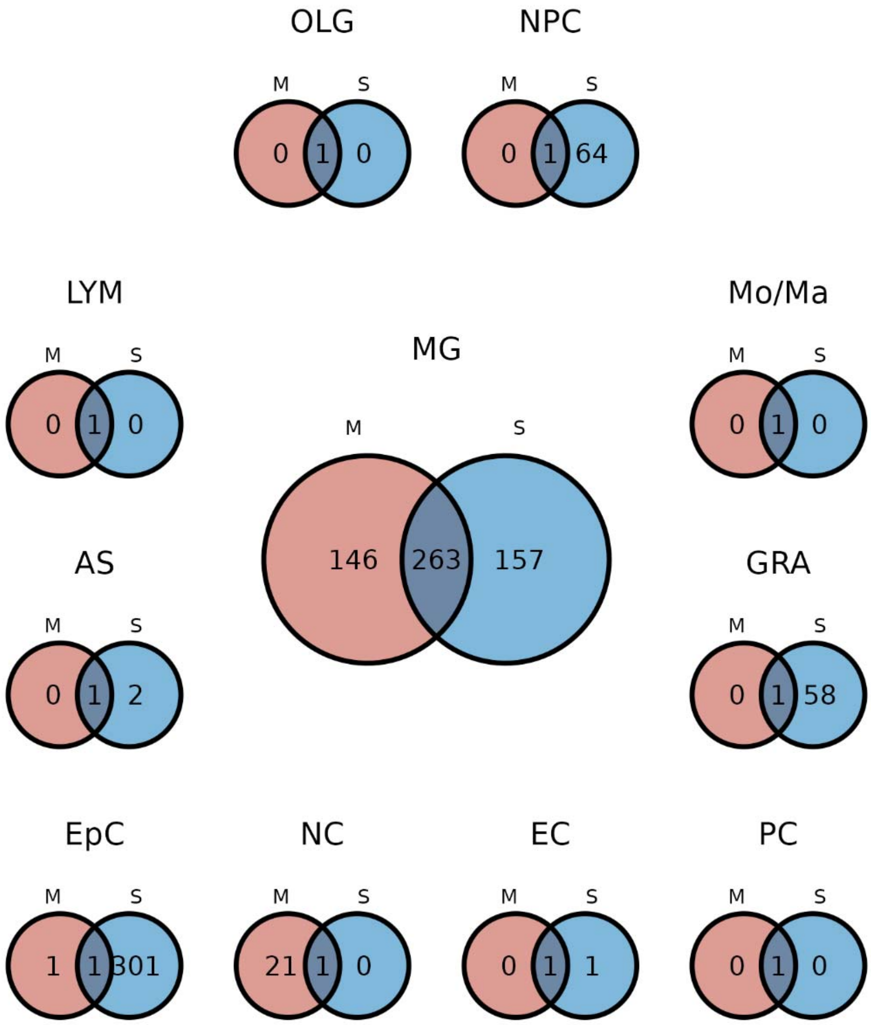

Figure S3. Venn diagram of P2ry12 correlated genes in tMCAO and Sham scRNA-seq data. This Venn diagram represents the overlap of genes that are positively correlated with P2ry12 in two experimental groups: tMCAO and Sham. The correlation coefficient was calculated using scRNA-seq data and was considered significant if its absolute value was greater than 0.6. The diagram shows the number of genes that are unique to each group as well as the genes that overlap between the groups. M, tMCAO; S, Sham; LYM, lymphocytes; OLG, oligodendrocytes; MG, microglia; NPC, neural progenitor cells; Mo/Ma, monocytes/macrophages; NC, neurons; AS, astrocytes; GRA, granulocytes; EpC, ependymal cells; EC, endothelial cells; PC, perivascular cells.

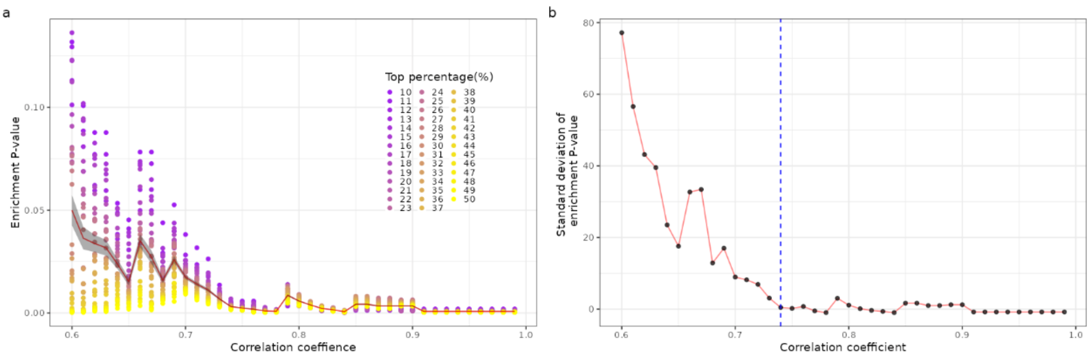

Figure S4. Determining the Optimal Correlation Coefficient for Microglia-Specific P2ry12 Genes (MSPGs). (**a**) The panel shows the results of a minimum-hypergeometric test evaluating the enrichment of sets of P2ry12 positive correlation genes obtained using different consecutive correlation coefficient cut-off thresholds (0.60, 0.61, …, 0.99) in the bulk-seq data-derived rank list obtained using different consecutive top percentage cut-off thresholds (10%, 11%, …, 50%). The mean enrichment P-value is displayed as a red curve, and the standard error of the mean is represented by the grey ribbon. (**b**) The panel displays the standard deviation of the sets of enrichment P-values derived from different correlation coefficients. The standard deviation is indicated by a black dot, and the optimal correlation coefficient of 0.74 is indicated by a dashed blue line on the X-axis.

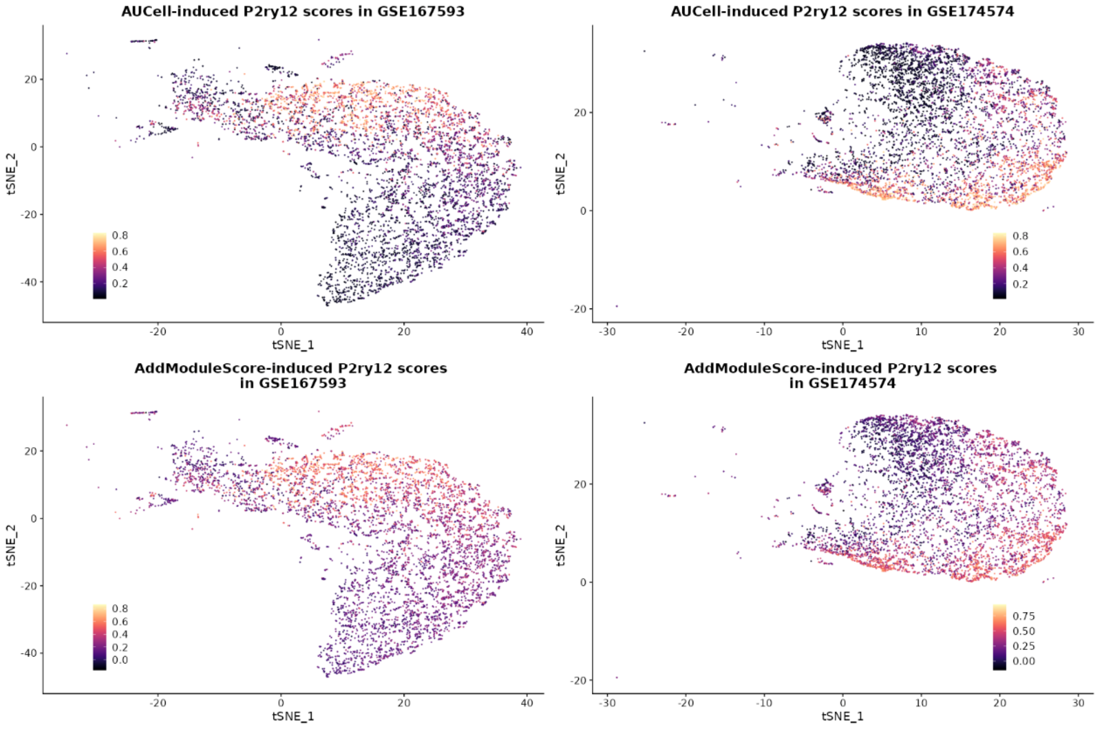

Figure S5. t-SNE plots demonstrating the diverse expression patterns of MSPGs-induced scores generated using either AddModuleScore or AUCell in microglial cells.

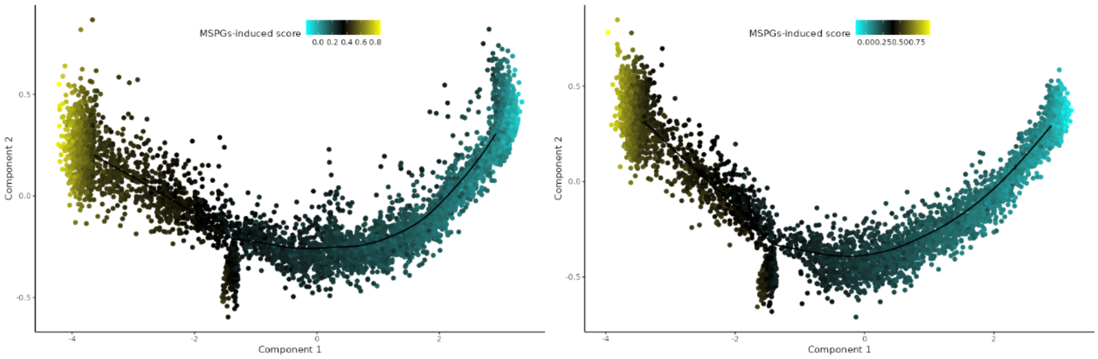

Figure S6. Projection of the MSPGs-induced scores computed by AUCell onto the trajectory of microglial cells.

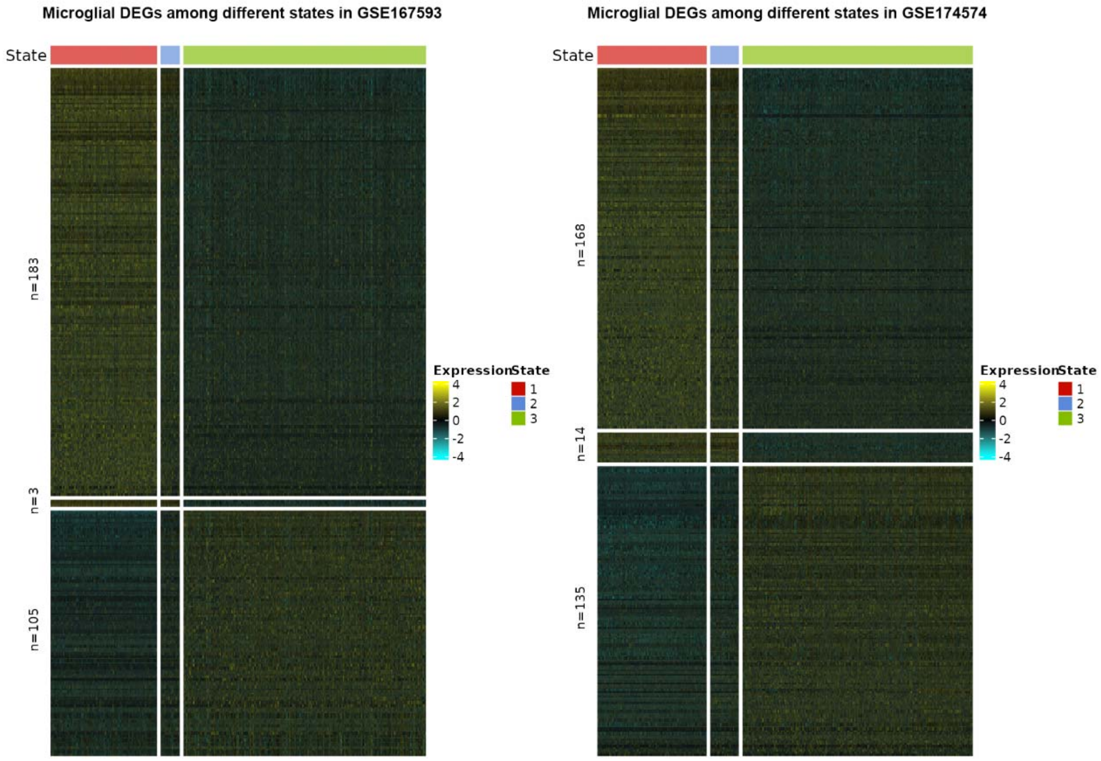

Figure S7. Differentially Expressed Genes in Different Microglial States. These heatmaps display the differentially expressed genes (DEGs) that are up-regulated in three distinct microglial states based on P2ry12 expression levels. The three microglial states are labeled as State 1 (high-P2ry12 microglia), State 2 (median-P2ry12 microglia), and State 3 (ow-P2ry12 microglia). The number of up-regulated DEGs is indicated by "n".

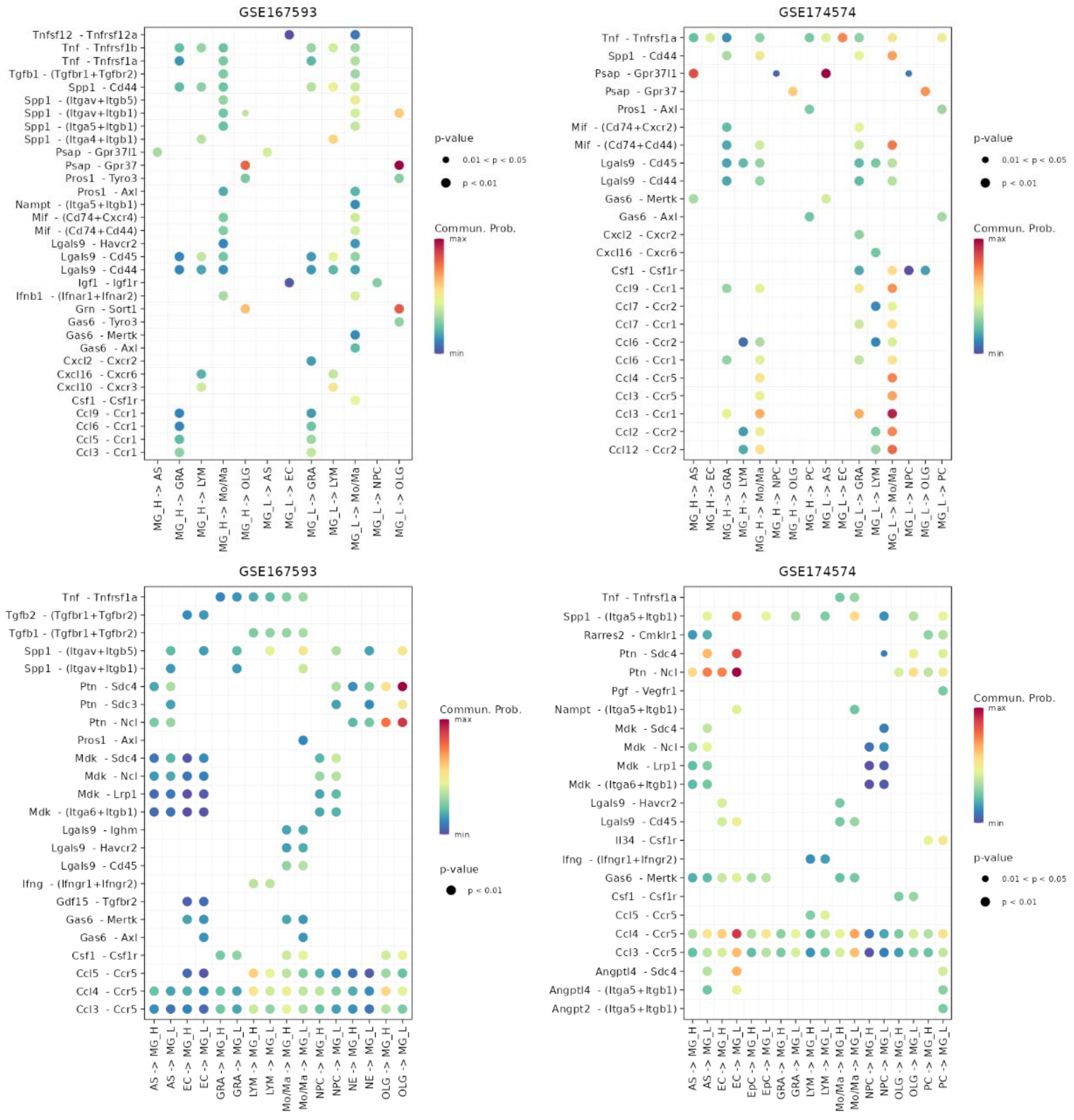

Figure S8. Cell-cell Interactions between Microglia and Other Cortical Populations. These bubble plots provide a visual representation of the cell-cell interactions between low-P2ry12 and high-P2ry12 microglia and other cortical populations. The first row of the plots shows the interactions that are induced by the up-regulated ligands in low-P2ry12 and high-P2ry12 microglia, while the second row shows the interactions induced by the up-regulated receptors. MG_L, low-P2ry12 microglia; MG_H, high-P2ry12 microglia; LYM, lymphocytes; OLG, oligodendrocytes; NPC, neural progenitor cells; Mo/Ma, monocytes/macrophages; NC, neurons; AS, astrocytes; GRA, granulocytes; EpC, ependymal cells; EC, endothelial cells; PC, perivascular cells.

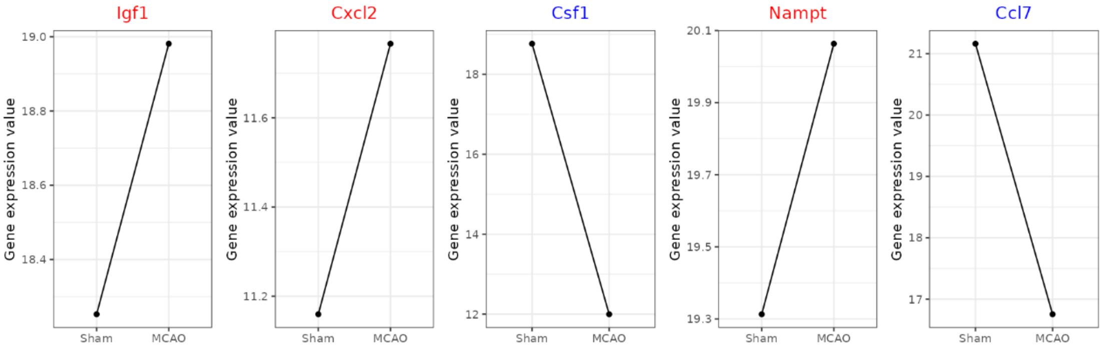

Figure S9. Expression trends of up-regulated ligand-encoding genes in microglia with low p2ry12 expression. These line plots display the expression tendencies of up-regulated ligand-encoding genes in low-P2ry12 microglia in integrated bulk-seq data. The genes that pass a specified test are indicated in red, while genes that do not pass the test are indicated in blue.

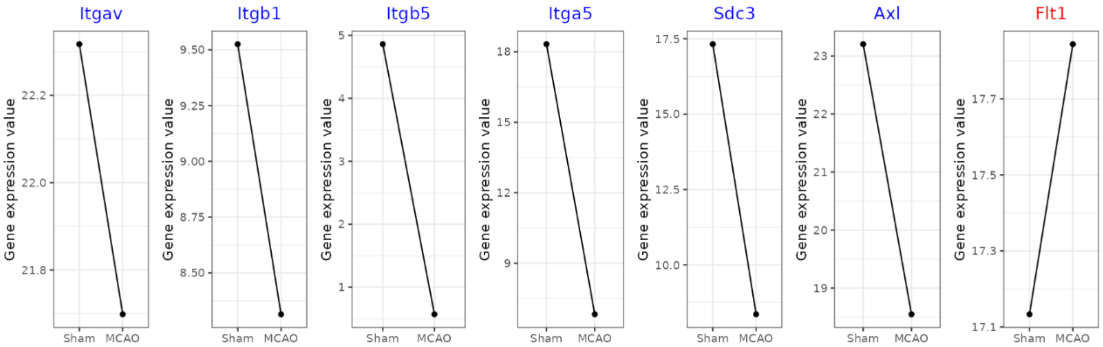

Figure S10. Expression trends of up-regulated receptor-encoding genes in microglia with low p2ry12 expression. These line plots display the expression tendencies of up-regulated receptor-encoding genes in low-P2ry12 microglia in integrated bulk-seq data. The genes that pass a specified test are indicated in red, while genes that do not pass the test are indicated in blue.

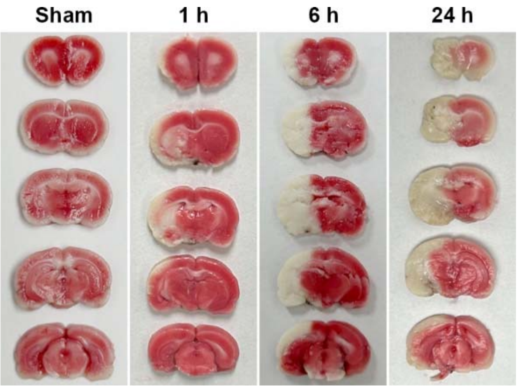

Figure S11. shows 2,3,5-tetraphenyltetrazolium chloride (TTC) staining results of rat brain slices following a 1-hour occlusion of the left external carotid artery and sham surgery. The images were taken at 1 hour, 6 hours, and 24 hours after reperfusion. The white color areas indicate regions of brain injury, while the red color areas represent normal brain tissue.

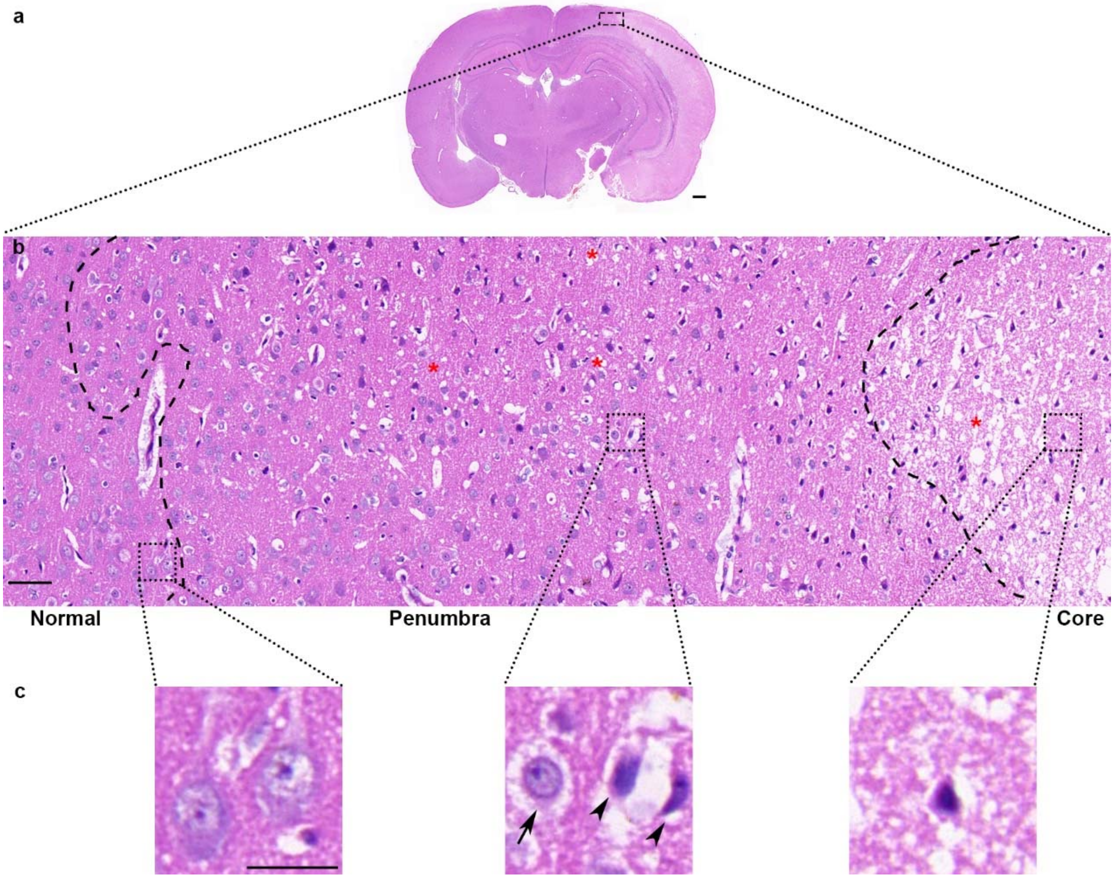

Figure S12. Morphological variation of rat brain undergoing IRI in HE staining. (**a**) Coronal section of the brain shows a pale region indicating an ischemic core lesion. (**b**) A zoom-in of the area indicated by the black rectangle in (**a**) shows three distinct regions: normal, penumbra, and ischemic core, separated by two dashed curves. Red asterisks indicate neuropil vacuolation. (**c**) Zoom-ins of representative neurons in the three regions in (**b**). The left image shows two neurons with large nuclei, fine chromatin, and obvious nucleoli. The middle image shows a relatively normal neuron (arrow) and two necrotic neurons (arrowheads) with peri-cellular halos. The right image shows a shrunken necrotic neuron within the neuropil with vacuolation changes. (Scale bars: 500μm for (**a**), 50μm for (**b**), and 20μm for (**c**))

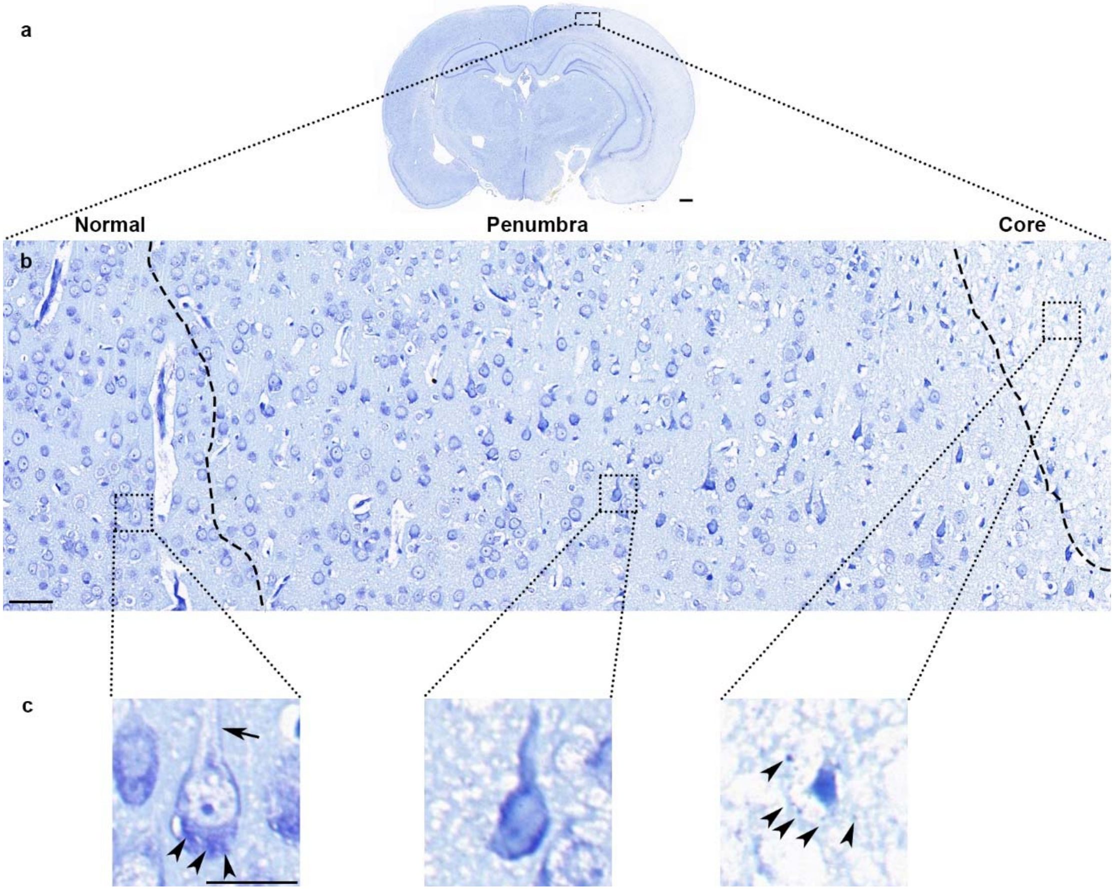

Figure S13. Morphological variation of rat brain undergoing IRI in Nissl staining. (**a**) Coronal section of the brain shows a pale region indicating an ischemic core lesion. (**b**) A zoom-in of the area indicated by the black rectangle in (**a**) shows three distinct regions: normal, penumbra, and ischemic core, separated by two dashed curves. (**c**) Zoom-ins of representative neurons in the three regions in (b). The left image shows a neuron at the center with a large nucleus, leptochromatin, and an obvious nucleolus, with clumps of Nissl substrates deposited in the perikarya (arrowheads) but not in the axon (arrow). The middle image shows a necrotic neuron with a shrunken nucleus and an inconspicuous nucleolus, with the deposition of Nissl substrates in both the perikarya and axon. The right image shows a completely shrunken necrotic neuron embedded in vacuolated neuropil, accompanied by surrounding necrotic debris (arrowheads). (Scale bars: 500μm for (**a**), 50μm for (**b**), and 20μm for (**c**))

Table S1 Imputed and pre-imputed P2ry12 expression value in populations of GSE167593

Population	Group	Data_type	Min.	1stQ	Median	3stQ	Max.	Mean	DR
Lymphocytes	tMCAO	Pre_imputed	0.0000	0.0000	0.0000	0.0000	2.7891	0.0616	0.9534
Lymphocytes	tMCAO	Imputed	0.0000	0.0000	0.0000	0.0000	2.7891	0.0661	0.9453
Lymphocytes	Sham	Pre_imputed	0.0000	0.0000	0.0000	0.0000	2.9037	0.0556	0.9509
Lymphocytes	Sham	Imputed	0.0000	0.0000	0.0000	0.0000	2.9037	0.0608	0.9425
OLG	tMCAO	Pre_imputed	0.0000	0.0000	0.0000	0.0000	2.9574	0.0523	0.9548
OLG	tMCAO	Imputed	0.0000	0.0000	0.0000	0.0000	2.9574	0.0551	0.9489
OLG	Sham	Pre_imputed	0.0000	0.0000	0.0000	0.0000	2.8124	0.0607	0.9471
OLG	Sham	Imputed	0.0000	0.0000	0.0000	0.0000	2.8124	0.0640	0.9386

Microglia	tMCAO	Pre_imputed	0.0000	0.0000	1.1238	2.0184	4.5488	1.2048	0.2936
Microglia	tMCAO	Imputed	0.0000	0.7766	1.2509	2.0915	3.9589	1.4334	0.1181
Microglia	Sham	Pre_imputed	0.0000	0.0000	1.3502	2.1378	3.7950	1.2965	0.2718
Microglia	Sham	Imputed	0.0000	0.8862	1.5145	2.2821	3.7794	1.5766	0.0911
NPC	tMCAO	Pre_imputed	0.0000	0.0000	0.0000	0.0000	3.4526	0.0528	0.9600
NPC	tMCAO	Imputed	0.0000	0.0000	0.0000	0.0000	3.4526	0.0558	0.9521
NPC	Sham	Pre_imputed	0.0000	0.0000	0.0000	0.0000	1.6568	0.0370	0.9601
NPC	Sham	Imputed	0.0000	0.0000	0.0000	0.0000	1.6568	0.0391	0.9553
Mo/Ma	tMCAO	Pre_imputed	0.0000	0.0000	0.0000	0.3509	2.3943	0.1840	0.7109
Mo/Ma	tMCAO	Imputed	0.0000	0.0000	0.0000	0.4474	2.3916	0.2195	0.6558
Mo/Ma	Sham	Pre_imputed	0.0000	0.0000	0.0000	0.3106	3.5027	0.1885	0.7320
Mo/Ma	Sham	Imputed	0.0000	0.0000	0.0000	0.4630	2.8272	0.2257	0.6688
Neurons	tMCAO	Pre_imputed	0.0000	0.0000	0.0000	0.0000	2.4602	0.0284	0.9811
Neurons	tMCAO	Imputed	0.0000	0.0000	0.0000	0.0000	2.4602	0.0344	0.9717
Neurons	Sham	Pre_imputed	0.0000	0.0000	0.0000	0.0000	2.1246	0.0649	0.9360
Neurons	Sham	Imputed	0.0000	0.0000	0.0000	0.0000	1.7521	0.0645	0.9244
Astrocytes	tMCAO	Pre_imputed	0.0000	0.0000	0.0000	0.0000	3.1547	0.0963	0.9512
Astrocytes	tMCAO	Imputed	0.0000	0.0000	0.0000	0.0000	3.1547	0.1150	0.9268
Astrocytes	Sham	Pre_imputed	0.0000	0.0000	0.0000	0.0000	3.0054	0.1309	0.9368
Astrocytes	Sham	Imputed	0.0000	0.0000	0.0000	0.0000	3.0054	0.1097	0.9368
Granulocytes	tMCAO	Pre_imputed	0.0000	0.0000	0.0000	0.0000	2.0146	0.0846	0.9388
Granulocytes	tMCAO	Imputed	0.0000	0.0000	0.0000	0.0000	2.0146	0.0968	0.9082
Granulocytes	Sham	Pre_imputed	0.0000	0.0000	0.0000	0.0000	3.0995	0.1084	0.9340
Granulocytes	Sham	Imputed	0.0000	0.0000	0.0000	0.0000	3.0995	0.1339	0.8924
Ependymal cells	tMCAO	Pre_imputed	0.0000	0.0000	0.0000	0.0000	2.3425	0.0545	0.9402
Ependymal cells	tMCAO	Imputed	0.0000	0.0000	0.0000	0.0000	2.3425	0.0572	0.9402
Ependymal cells	Sham	Pre_imputed	0.0000	0.0000	0.0000	0.0000	0.8065	0.0113	0.9821
Ependymal cells	Sham	Imputed	0.0000	0.0000	0.0000	0.0000	0.8941	0.0121	0.9821

DR, dropout ratio; OLG, oligodendrocytes; NPC, neural progenitor cells; Mo/Ma, monocytes/macrophages.

Table S2 Imputed and pre-imputed P2ry12 expression value in populations of GSE174574

Population	Group	Data_type	Min.	1stQ	Median	3stQ	Max.	Mean	DR
Endothelial cells	tMCAO	Pre_imputed	0.0000	0.0000	0.0000	0.0000	2.6846	0.0785	0.9484
Endothelial cells	tMCAO	Imputed	0.0000	0.0000	0.0000	0.0000	2.6846	0.0881	0.9371
Endothelial cells	Sham	Pre_imputed	0.0000	0.0000	0.0000	0.0000	3.3430	0.3275	0.8072
Endothelial cells	Sham	Imputed	0.0000	1.9745	2.0474	2.1211	3.3575	1.9099	0.0741
Mo/Ma	tMCAO	Pre_imputed	0.0000	0.0000	0.0000	0.0000	2.7679	0.1790	0.8500
Mo/Ma	tMCAO	Imputed	0.0000	0.0000	0.0000	0.0000	2.5499	0.2578	0.7586
Mo/Ma	Sham	Pre_imputed	0.0000	0.0000	0.0000	1.3176	3.3648	0.5981	0.6106
Mo/Ma	Sham	Imputed	0.0000	2.0971	2.2967	2.4288	3.7637	2.1363	0.0586
Ependymal cells	tMCAO	Pre_imputed	0.0000	0.0000	0.0000	0.0000	2.0568	0.0752	0.9311
Ependymal cells	tMCAO	Imputed	0.0000	0.0000	0.0000	0.0000	2.0568	0.0778	0.9281
Ependymal cells	Sham	Pre_imputed	0.0000	0.0000	0.0000	0.7735	3.1999	0.3740	0.6667

Ependymal cells	Sham	Imputed	0.0000	2.0216	2.0803	2.1339	3.9304	2.0531	0.0192
Microglia	tMCAO	Pre_imputed	0.0000	0.0000	1.2931	2.3014	4.5363	1.3067	0.3758
Microglia	tMCAO	Imputed	0.0000	1.0285	1.6136	2.5207	3.7574	1.6765	0.1509
Microglia	Sham	Pre_imputed	0.0000	3.2700	3.6563	3.9625	4.8844	3.5112	0.0149
Microglia	Sham	Imputed	2.2024	4.4753	4.6472	4.7836	5.2713	4.5465	0.0000
OLG	tMCAO	Pre_imputed	0.0000	0.0000	0.0000	0.0000	2.0628	0.0869	0.9232
OLG	tMCAO	Imputed	0.0000	0.0000	0.0000	0.0000	2.0628	0.0946	0.9149
OLG	Sham	Pre_imputed	0.0000	0.0000	0.0000	0.9268	1.8846	0.3744	0.6536
OLG	Sham	Imputed	0.0000	2.0433	2.0980	2.1460	3.0231	2.0830	0.0078
Astrocytes	tMCAO	Pre_imputed	0.0000	0.0000	0.0000	0.0000	2.7065	0.1322	0.9024
Astrocytes	tMCAO	Imputed	0.0000	0.0000	0.0000	0.0000	2.7065	0.1471	0.8820
Astrocytes	Sham	Pre_imputed	0.0000	0.0000	0.0000	1.0830	3.2895	0.4314	0.7181
Astrocytes	Sham	Imputed	0.0000	2.0435	2.1072	2.1712	4.0630	2.0654	0.0342
Perivascular cells	tMCAO	Pre_imputed	0.0000	0.0000	0.0000	0.0000	3.0048	0.0680	0.9505
Perivascular cells	tMCAO	Imputed	0.0000	0.0000	0.0000	0.0000	3.0048	0.0756	0.9426
Perivascular cells	Sham	Pre_imputed	0.0000	0.0000	0.0000	0.0000	2.6871	0.3472	0.7635
Perivascular cells	Sham	Imputed	0.0000	1.9822	2.0523	2.1270	3.5948	1.9765	0.0428
Lymphocytes	tMCAO	Pre_imputed	0.0000	0.0000	0.0000	0.0000	1.9756	0.1021	0.9240
Lymphocytes	tMCAO	Imputed	0.0000	0.0000	0.0000	0.0000	2.1165	0.1422	0.8815
Lymphocytes	Sham	Pre_imputed	0.0000	0.0000	0.0000	0.0000	2.7594	0.3470	0.7636
Lymphocytes	Sham	Imputed	0.0000	1.8589	1.9797	2.0617	3.4738	1.6990	0.1455
Granulocytes	tMCAO	Pre_imputed	0.0000	0.0000	0.0000	0.0000	2.5300	0.1431	0.9163
Granulocytes	tMCAO	Imputed	0.0000	0.0000	0.0000	0.0000	2.4011	0.2007	0.8536
Granulocytes	Sham	Pre_imputed	0.0000	0.0000	0.0000	0.9004	3.8773	0.5126	0.6711
Granulocytes	Sham	Imputed	1.8637	2.0888	2.1854	2.2799	4.5841	2.2513	0.0000
NPC	tMCAO	Pre_imputed	0.0000	0.0000	0.0000	0.0000	2.0180	0.1979	0.8298
NPC	tMCAO	Imputed	0.0000	0.0000	0.0000	0.8688	2.0180	0.3377	0.7021
NPC	Sham	Pre_imputed	0.0000	0.0000	0.0000	0.4344	2.7707	0.5200	0.7500
NPC	Sham	Imputed	0.0000	2.0621	2.0986	2.2279	3.1381	2.1027	0.0833

DR, dropout ratio; OLG, oligodendrocytes; Mo/Ma, monocytes/macrophages; NPC, neural progenitor cells.

Table S3 P2ry12 correlation genes in microglia of single-cell expression profile

Genename	Corr in Sham	Corr in tMCAO
P2ry12	1	1
Selplg	0.936572763	0.965191924
Siglech	0.907457765	0.949658181
Slc2a5	0.907420451	0.926555751
Tmem119	0.903587485	0.962807635
Vsir	0.89476639	0.938958088
Crybb1	0.89123398	0.884848677
Gpr34	0.889668529	0.891042553
0610040J01Rik	0.883591958	0.893857937
P2ry13	0.883210997	0.918135128

Ptgs1	0.88214684	0.929760397
Kcnk12	0.880605373	0.884981838
Susd3	0.878897565	0.931351761
Cmtm6	0.878726748	0.824526497
Serinc3	0.876088553	0.758556579
Lpcat2	0.874220909	0.930429071
Fam105a	0.872088006	0.862261045
Bbs9	0.870737527	0.819122107
Upk1b	0.864976953	0.887091896
F11r	0.862922056	0.848178578
Hpgd	0.860109118	0.676445032
Entpd1	0.858043848	0.843492445
Cd164	0.856888856	0.649514312
Commd8	0.85565689	0.813833117
Slco2b1	0.855432999	0.814588615
Atp8a2	0.849792473	0.828694204
Agmo	0.8477917	0.830791291
Adgrg1	0.847627104	0.860620221
Gp9	0.847513235	0.885611149
Cysltr1	0.847404734	0.809098579
Gna15	0.847252816	0.858895988
Lrrc3	0.846020324	0.876556779
Tmem173	0.844601751	0.837333225
BC035044	0.843979417	0.852590259
Myl2	0.843977875	0.786650804
Tpst2	0.842318247	0.783129466
Rnase4	0.837100616	0.640508283
Ctc1	0.835909508	0.815553921
Pld4	0.835310218	0.73655391
Capn3	0.834336836	0.871755605
Csf1r	0.833180807	0.764226526
Kcnd1	0.830119673	0.862138846
Fgd2	0.827691725	0.836082761
Golm1	0.825952244	0.814023856
2610528A11Rik	0.825250642	0.830966877
Snn	0.823477173	0.846766095
Chst7	0.823091188	0.869575303
Garnl3	0.821279254	0.831839254
Pde3b	0.820544621	0.866517353
Tubgcp5	0.820146802	0.677235705
Sipa1	0.819074915	0.698714153
Tgfbr1	0.816750476	0.789415679
Cmtm7	0.814651253	0.766329243
Hnmt	0.813392192	0.832872154

Sult1a1	0.813252023	0.617841938
Snta1	0.810255357	0.782798559
St3gal5	0.810086956	0.879577187
Sall1	0.809220247	0.870401503
Hhex	0.809151576	0.690183214
Col27a1	0.807499629	0.823152625
Ogfrl1	0.80669422	0.663897222
Rogdi	0.804479867	0.845371338
St3gal6	0.80447602	0.69511085
Ppcdc	0.801255043	0.79873672
Cd33	0.797853865	0.754658846
Ctsf	0.797561164	0.771240722
Tnfrsf13b	0.795960663	0.757640338
Rps6ka1	0.795028131	0.801293931
A830008E24Rik	0.794501167	0.871778463
Bco2	0.793559848	0.838445712
Il16	0.78993762	0.718869278
Slc46a1	0.788803892	0.879213777
Rtn4rl1	0.788077622	0.809785769
Dapp1	0.786024001	0.756209036
Gm3739	0.785055843	0.810022915
Ifngr1	0.784643557	0.81690588
Tanc2	0.783240635	0.885557827
Arhgap5	0.782715643	0.772258229
Ivns1abp	0.780178952	0.662544508
Csmd3	0.779232886	0.822713938
Prkab1	0.774945341	0.752917811
Zfhx3	0.771835233	0.73664553
Nrm	0.770631196	0.713232034
Cxxc5	0.769655739	0.782698874
Liph	0.769490552	0.751668589
Csnk1e	0.767764152	0.751990171
Siglece	0.767515302	0.656335091
Scamp2	0.767134378	0.85517013
Cx3cr1	0.766833513	0.917192599
Prpsap2	0.764449406	0.691028687
Rp2	0.76287572	0.739339765
Il6ra	0.760593377	0.746912711
Gm16118	0.759485077	0.798221389
Ssh2	0.757858676	0.692773729
Zfp710	0.754856501	0.697303142
Mlph	0.753527711	0.768357048
Ikzf1	0.753502304	0.831106682
Bend6	0.751882431	0.789406064

Cst3	0.751144815	0.786811597
Cdh23	0.748860381	0.788024654
Ebf3	0.748762569	0.789750158
Hps4	0.748738019	0.777271162
Cep152	0.747152706	0.614308226
Elmo1	0.747058927	0.751272301
Slc29a3	0.746234512	0.805602482
Plcl2	0.745879421	0.815254242
Nuak1	0.745571085	0.69693309
Fscn1	0.744939456	0.814568012
Inpp5d	0.743944176	0.733617461
Tpbgl	0.743193315	0.811430217
Alox5ap	0.742764922	0.669707639
Hpgds	0.742409427	0.645934369
Rcsd1	0.74180872	0.621266574
Marcks	0.739683217	0.746948958
Abhd15	0.738853402	0.688284087
Srgap2	0.737172341	0.701829739
Mfng	0.736752505	0.793019637
Cask	0.736529138	0.671143021
Gtf2h2	0.73610284	0.811204687
Traf3ip3	0.735598873	0.779412823
Abi3	0.735213414	0.818071802
Bin2	0.734021875	0.853958702
Pld1	0.732263996	0.698172766
Nfam1	0.731320854	0.650454294
Lrba	0.731277987	0.798374387
Stambpl1	0.730884093	0.718249744
Sft2d1	0.728569774	0.72149448
Slco4a1	0.726133926	0.753341175
Gm3488	0.725780798	0.729283105
Icosl	0.725696567	0.662460612
Tmx4	0.725611708	0.645491743
I830077J02Rik	0.725492765	0.799542861
Sall3	0.724434985	0.807555113
Gal3st4	0.723694727	0.813929159
Laptm5	0.722365148	0.719290402
Lair1	0.721930302	0.877801532
Matk	0.721690737	0.778808809
Akirin2	0.720157216	0.605781105
Cd37	0.719991516	0.772357977
Rab39	0.717986015	0.625914831
Pag1	0.71681225	0.764527535
Epb41l2	0.716334235	0.774050723

Frmd4a	0.715153684	0.837586964
Cryl1	0.713523181	0.672108531
Unc93b1	0.711857899	0.662182762
Tspan18	0.709868336	0.728467283
Tmem135	0.709642235	0.720146185
Gpr155	0.709424914	0.743730339
Lactb	0.709278692	0.771042324
Serpinf1	0.709059099	0.620689406
Fam102b	0.706849177	0.75741867
Sgce	0.705583481	0.742976645
Arsk	0.705582665	0.692205805
Casp8	0.704119406	0.696505286
2310040G24Rik	0.701689923	0.637641213
Cttnbp2nl	0.701553434	0.749027447
Celf2	0.701269561	0.610650028
Cryba4	0.701186427	0.811481401
Arhgap22	0.701051666	0.730998792
Sparc	0.699602829	0.723932887
March1	0.699144231	0.777688827
B4galt4	0.699136518	0.695811152
Sash3	0.698507083	0.655559871
Glul	0.698097462	0.629803227
Mgat1	0.697793742	0.728518286
Gng10	0.695911913	0.785593712
Bid	0.694692827	0.71435766
P3h2	0.692949935	0.699338663
Fam212a	0.692540158	0.606983237
Whrn	0.690978286	0.744087924
C5ar2	0.690346397	0.600881577
Tlr12	0.689763143	0.78581416
Gm12166	0.688143996	0.618312608
Mef2c	0.688059928	0.60683749
Usp2	0.687297902	0.798819805
Gcnt1	0.686030141	0.709476571
Galnt12	0.683002425	0.853266477
Ecscr	0.680108334	0.878287679
Cd79b	0.678317756	0.75974717
Arsb	0.677614338	0.66005343
Armc3	0.674537742	0.680215397
Twf2	0.673866038	0.679045814
Gm3248	0.672997875	0.67571369
Pecr	0.672855678	0.746660906
Cd81	0.672287186	0.738184882
Slc46a3	0.671864078	0.605416708

Tspan14	0.671593792	0.771097266
Klhdc8b	0.670543183	0.707796066
Itgb5	0.66997469	0.708744014
Gm10605	0.668362281	0.63358901
Rassf5	0.668205828	0.709289624
Cnot8	0.66817136	0.671162881
Slc13a3	0.66790255	0.601983179
Mtdh	0.667417116	0.802195739
Pald1	0.666319181	0.616312725
Adrb1	0.665301146	0.634078118
Ifitm10	0.664996784	0.748418972
Khk	0.663951206	0.607654223
Usp21	0.662664038	0.716574109
Ccng2	0.662648487	0.787961744
Hsd17b11	0.661577364	0.681327465
Prkca	0.661118728	0.65876828
Smap2	0.660866063	0.772442661
Med12l	0.65929085	0.767885185
Trp53cor1	0.659151024	0.6808953
Dock10	0.658789633	0.679139904
BC037034	0.658529389	0.64482001
Rab3ip	0.657415869	0.669440017
Numb	0.657413915	0.740973895
Gm3636	0.654903916	0.782836365
Zbtb18	0.653559852	0.688388117
Papss1	0.65289333	0.722196311
Cd82	0.65287318	0.618098978
Ncf1	0.651968544	0.683312081
Rhoh	0.650654952	0.808703636
Fbrsl1	0.649616059	0.68490309
Tmem100	0.648816733	0.747876855
Kcnk6	0.648011346	0.73720484
Cyth4	0.647025002	0.669402966
Rnf13	0.646213148	0.669590419
Mgat4a	0.644910651	0.683908387
Csf3r	0.642379165	0.671211944
Mknk1	0.641100147	0.683609006
Mdfi	0.641001514	0.618636478
Hexb	0.638831618	0.802872947
Slc16a6	0.63828108	0.86066891
Sox4	0.637577321	0.729568314
Taz	0.635621243	0.715496788
Nav2	0.635006989	0.786994064
Ppfia4	0.634980174	0.774812567

Pdk1	0.634295471	0.707760904
Parvg	0.631207623	0.801799029
Oma1	0.630549747	0.727193547
Ankrd44	0.630206665	0.63393863
Csk	0.628403123	0.624609373
Sema4g	0.626932851	0.609107275
Tgif2	0.626692463	0.657264899
Fam49b	0.626173395	0.780830541
Olfml3	0.624130006	0.862677097
Zfp385a	0.624056948	0.607290271
1810011H11Rik	0.623940834	0.670964237
Cebpz	0.62362757	0.621105442
Gmip	0.622250458	0.632908258
Arhgap4	0.62197632	0.707367399
Ldhb	0.621307085	0.666042856
H2-DMa	0.619351389	0.602607069
Fcrl1	0.618006735	0.780315611
Soga1	0.615527512	0.650634567
Scoc	0.613020139	0.801930217
Man1a2	0.61203896	0.705082003
Vrk2	0.610639861	0.619901895
Kif21b	0.609239403	0.611866475
Tnfrsf21	0.607971986	0.733008035
Acox3	0.60643261	0.630238377
Tmem52	0.605664459	0.715672265
Tmem68	0.605607685	0.612013289
Accs	0.605387848	0.76284247
Arhgap27	0.605005773	0.690352959
Pnp	0.604965522	0.608147292
Slc44a2	0.603442718	0.654164916
Dip2b	0.603274856	0.653447
Pou2f2	0.600941357	0.727894023
Rab7b	-0.656989619	-0.661593453

Corr, correlation.

Appendix SA. Evaluation of Single-Cell Annotation Tools for Mouse Cerebral Cortical Cells

Annotating single-cell transcriptomic maps involves labeling cell types or even subtypes with gene expression matrix. This process helps to understand the diversity of cell types and the underlying biological processes in a tissue or organism. Manual and automatic annotation methods are the two main categories of annotation techniques used for single-cell transcriptomic maps [1]. Manual annotation involves expert biologists who review the data and label cells based on their gene expression profiles. This method is time-consuming but provides high accuracy, as it is based on expert knowledge and can uncover novel cell types. However, manual annotation is limited in its scalability, as it requires significant resources and expertise. Automatic annotation, on the other hand, is performed using computational methods, such as clustering and gene set enrichment analysis. These methods process the data and label cells based on the similarity of their gene expression profiles to known cell types. This method is faster and more scalable, as it can process large amounts of data in a short amount of time. However, automatic annotation is limited in its accuracy, as it relies on the quality of the data and the algorithms used. Nevertheless, automatic annotation methods are typically more effective for annotating major cell types [2].

There are two main methods for automatic single-cell annotation: marker-based and reference-based [3]. Marker-based annotation relies on the identification of known cell type-specific markers, such as genes or proteins, to assign cell types

to individual cells. Reference-based annotation, on the other hand, compares the molecular and functional properties of individual cells to a reference set of annotated cells to assign cell types. Here, we utilized multiple commonly used automatic annotation tools to annotate the mouse cerebral cortical scRNA-seq data from SeuratData [4] (https://seurat.nygenome.org/azimuth/demo_datasets/allen_mop_2020.rds). The scRNA-seq data consists of various cell types, including astrocytes, endothelial cells, microglia, neurons, oligodendrocytes, and perivascular cells. The annotated results were integrated in various combinations and compared to expert annotations within the data to determine the optimal approach for annotating the mouse cerebral cortical scRNA-seq data used in the main text. The tools we used for automatic annotation are shown in the following Table S4.

Table S4 summary of automatic annotation tools

**Type**	**Tool**	**Reference Source**
Reference based	scmap-cluster [5]<br>scmap-cell [5]<br>singleR [6]	celldex
Marker based	SCINA [7]	PanglaoDB [8]<br>Cellmarker [9]

We employed three reference-based tools to annotate the mouse cerebral cortical scRNA-seq data. The tool "scmap-cluster" was used to compute the

centroids of each cell type and project them onto the query data. The other two reference-based tools project the cells of the input dataset to the individual cells of the reference. The reference scRNA-seq data used in this analysis was derived from a mouse motor dataset provided by the "celldex" package. Additionally, a marker-based tool named "SCINA" was used, and it employed two marker reference data sources: "PanglaoDB" (available at https://panglaodb.se/index.html) (accessed on 14 February 2023) and "Cellmarker" (available at http://yikedaxue.slwshop.cn/) (accessed on 14 February 2023).

The results obtained from these five methods were randomly combined to produce 31 different annotation results. The final annotated label for each cell was assigned based on the most common label across tools. Cells that lacked a clear cell type (i.e., different tools showed different results) were labeled as "ambiguous" in the combination.

To assess the accuracy of the automated annotations, the match rate between the cell types inferred by the different tools and the cell types provided by expert annotations was calculated in two ways: (1) match rate without ambiguous cells, which is calculated by excluding cells labeled as ambiguous, and (2) total match rate, which includes all cells. Of the 31 match results without ambiguous cells, 18 (58%) had an extremely high match rate exceeding 97%. Of the 31 total match results, 26 (84%) had a high match rate exceeding 90% (as shown in Table S5). In this study, as described in the main text, the downstream analysis was performed

using cells with clearly defined cell types. Therefore, we place particular emphasis on the match rate without ambiguous cells. Based on the analysis of the cell type match rates, a combination of SingleR, scmap-cell, and Cellmarker-based SCINA was found to be the optimal combination of automatic annotation tools for mouse cerebral cortical cells.

Table S5. Match rate between tool-derived and expert-provided cell types

**Tool Names**	**MR**	**tMR**
Sc-R-Sm	0.9799	0.9649
Sc-R-Sp	0.9799	0.9647
Sl-R-Sm-Sp	0.9799	0.9621
Sc-Sm	0.9794	0.9305
Sc-R-Sm-Sp	0.9794	0.9636
Sl-Sm	0.9791	0.9248
R-Sm	0.9784	0.9336
Sl-R-Sp	0.9782	0.9643
Sl-R-Sm	0.9778	0.9651
R-Sm-Sp	0.9764	0.9511
Sl-R	0.9762	0.9571
Sc-R	0.9761	0.9598
Sl-Sm-Sp	0.9761	0.9518
Sc-Sm-Sp	0.9757	0.9513
Sl-Sp	0.9755	0.7654
Sc-Sp	0.9750	0.7716
R-Sp	0.9732	0.7741
Sm-Sp	0.9709	0.7600
Sl-Sc-R-Sm-Sp	0.9680	0.9668
R	0.9679	0.9589
Sl-Sc-Sm-Sp	0.9679	0.9621
Sl-Sc-R-Sm	0.9676	0.9640
Sl-Sc-R-Sp	0.9671	0.9635
Sl-Sc-Sm	0.9664	0.9658
Sl-Sc-Sp	0.9661	0.9652
Sl-Sc	0.9659	0.9534
Sl-Sc-R	0.9642	0.9610
Sc	0.9625	0.9598
Sl	0.9571	0.9571
Sm	0.9358	0.9358
Sp	0.7762	0.7762

Sl, scmap-cluster; Sc, scmap-cell; R, singleR; Sp, SCINA based on PanglaoDB; Sm, SCINA based on Cellmarker; MR, match rate without ambiguous cells; tMR, total match rate.

1. Pasquini, G.; Rojo Arias, J. E.; Schafer, P.; Busskamp, V., Automated methods for cell type annotation on scRNA-seq data. *Comput Struct Biotechnol J* **2021,** *19*, 961-969.
2. Clarke, Z. A.; Andrews, T. S.; Atif, J.; Pouyabahar, D.; Innes, B. T.; MacParland, S. A.; Bader, G. D., Tutorial: guidelines for annotating single-cell transcriptomic maps using automated and manual methods. *Nat Protoc* **2021,** *16*, 2749-2764.
3. Chen, Y.; Zhang, S., Automatic Cell Type Annotation Using Marker Genes for Single-Cell RNA Sequencing Data. *Biomolecules* **2022,** *12*.
4. Yao, Z.; Liu, H.; Xie, F.; Fischer, S.; Adkins, R. S.; Aldridge, A. I.; Ament, S. A.; Bartlett, A.; Behrens, M. M.; Van den Berge, K.; Bertagnolli, D.; de Bezieux, H. R.; Biancalani, T.; Booeshaghi, A. S.; Bravo, H. C.; Casper, T.; Colantuoni, C.; Crabtree, J.; Creasy, H.; Crichton, K.; Crow, M.; Dee, N.; Dougherty, E. L.; Doyle, W. I.; Dudoit, S.; Fang, R.; Felix, V.; Fong, O.; Giglio, M.; Goldy, J.; Hawrylycz, M.; Herb, B. R.; Hertzano, R.; Hou, X.; Hu, Q.; Kancherla, J.; Kroll, M.; Lathia, K.; Li, Y. E.; Lucero, J. D.; Luo, C.; Mahurkar, A.; McMillen, D.; Nadaf, N. M.; Nery, J. R.; Nguyen, T. N.; Niu, S. Y.; Ntranos, V.; Orvis, J.; Osteen, J. K.; Pham, T.; Pinto-Duarte, A.; Poirion, O.; Preissl, S.; Purdom, E.; Rimorin, C.; Risso, D.; Rivkin, A. C.; Smith, K.; Street, K.; Sulc, J.; Svensson, V.; Tieu, M.; Torkelson, A.; Tung, H.; Vaishnav, E. D.; Vanderburg, C. R.; van Velthoven, C.; Wang, X.; White, O. R.; Huang, Z. J.; Kharchenko, P. V.; Pachter, L.; Ngai, J.; Regev, A.; Tasic, B.; Welch, J. D.; Gillis, J.; Macosko, E. Z.; Ren, B.; Ecker, J. R.; Zeng, H.; Mukamel, E. A., A transcriptomic and epigenomic cell atlas of the mouse primary motor cortex. *Nature* **2021,** *598*, 103-110.
5. Kiselev, V. Y.; Yiu, A.; Hemberg, M., scmap: projection of single-cell RNA-seq data across data sets. *Nat Methods* **2018,** *15*, 359-362.
6. Aran, D.; Looney, A. P.; Liu, L.; Wu, E.; Fong, V.; Hsu, A.; Chak, S.; Naikawadi, R. P.; Wolters, P. J.; Abate, A. R.; Butte, A. J.; Bhattacharya, M., Reference-based analysis of lung single-cell sequencing reveals a transitional profibrotic macrophage. *Nat Immunol* **2019,** *20*, 163-172.
7. Zhang, Z.; Luo, D.; Zhong, X.; Choi, J. H.; Ma, Y.; Wang, S.; Mahrt, E.; Guo, W.; Stawiski, E. W.; Modrusan, Z.; Seshagiri, S.; Kapur, P.; Hon, G. C.; Brugarolas, J.; Wang, T., SCINA: A Semi-Supervised Subtyping Algorithm of Single Cells and Bulk Samples. *Genes (Basel)* **2019,** *10*.
8. Franzen, O.; Gan, L. M.; Bjorkegren, J. L. M., PanglaoDB: a web server for exploration of mouse and human single-cell RNA sequencing data. *Database (Oxford)* **2019,** *2019*.
9. Zhang, X.; Lan, Y.; Xu, J.; Quan, F.; Zhao, E.; Deng, C.; Luo, T.; Xu, L.; Liao, G.; Yan, M.; Ping, Y.; Li, F.; Shi, A.; Bai, J.; Zhao, T.; Li, X.; Xiao, Y., CellMarker: a manually curated resource of cell markers in human and mouse. *Nucleic Acids Res* **2019,** *47*, D721-D728.
